# Periscope

**Published:** 1887-06

**Authors:** 


### PERISCOPE.

### SURGERY.

**Ephemeral Glycosuria in Surgical Practice.**—Dr. Redard has published a paper on this subject in the *Revue de Chirurgie*, 1866, Nos. 9 and 10.

He states that ephemeral glycosuria frequently appears in surgical practice: it is in some instances accompanied by ephemeral albuminuria; very often the sugar is replaced by albumen, and *vice versâ*. The condition has been noticed after slight injuries; but more frequently, in fact almost constantly, in phlegmonous and gangrenous affections, anthrax, lymphangitis, and erysipelas. The sugar usually appears just as pus is about to form, and when the temperature of the body is suddenly elevated. None of these patients presented glycosuria or other symptoms of diabetes after the cure of the surgical affection, nor had they a previous diabetic history. To account for the appearance of sugar in the urine at certain times, the author suggests that the impression produced at the periphery by a cutaneous inflammation, or some other local morbid condition, might be transmitted to the vaso-motor fibres contained in the splanchnic nerves, and so cause hyperæmia of certain organs, especially the liver and lungs, and accelerate the transformation of glycogen into sugar, thus giving rise to glycosuria.—*London Medical Record*, Dec., 1886.

**Chronic Serous Synovitis. Hydrarthrus.**—Dr. Edward Von Donhoff, in a paper read before the Louisville Medical Society, states that cases of hydrarthrus do not occur as frequently as those of fungous synovitis. They are distinguished from the latter, as in nowise tending to spontaneous pyogenesis; this phenomenon only occurring after repeated irritation or injury. Chronic hydrarthrus consists in a slow accumulation of thin synovial fluid, associated with very slight structural changes in the synovial membrane, which becomes only a little thickened, and is marked by an increase of connective tissue without any increase of vascularity. The synovial tufts are elongated, and in their apices the vessels become more tortuous. The granulation-changes due to serous and plastic infiltration are absent.

The origin of chronic dropsies of joints is ordinarily attributed to cold or traumatism; it is often the remnant of former articular trouble; is chronic from its inception, and remains so. The disease is, when unmodified by unavoidable circumstance or meddlesome surgery, a harmless inconvenience, overcome by judicious management, except in old subjects of lessened general vitality. The treatment may be described briefly thus: Rest; the promotion of absorption of effused fluids by pressure, local and general; tonic and alterative remedies, constitute the conservative treatment. Aspiration, followed by fixed dressing, and ice-cold water irrigation, represents the intermediate operative course. While free incision and evacuation, followed by hermetic sealing, fixed dressing and strict antiphlogistic regimen, make up the heroic procedure, permissible in rare instances of failure of the less formidable plans, or in cases where accident or mischievous ignorance has invited purulentiform alteration of the milder condition. Dr. Von Donhoff refers to the treatment by iodine injections into the joint, recommended by Boinet and Ricord, as impracticable and dangerous to life and limb, from its great liability to convert a simple catarrhal into a violent suppurating condition.—*American Practitioner and News*, April 2nd, 1887.

[No reference is made to the combined treatment of injections and subsequent free drainage.]

**Rectal Examinations in Morbus Coxæ.**—Dr. Arnold Schmitz, in the *Centralblatt für Chirurgie*, No. 11, 1886, discusses the above with respect to its diagnostic worth. The object of this examination is the palpation of the inner surface of the acetabulum through the rectum. The author reported three cases in which he had used the method, as follows:

(1) A boy, æt. 3, with an abscess over the trochanter, free movement of the hip-joint under narcosis, and some prominence of the lumbar spines. On rectal examination, a distinct swelling, prominent but not fluctuating, was felt in the acetabular region. This made the diagnosis of acetabular coxitis manifest. The subsequent course of the disease confirmed the diagnosis.

(2) A 5-year-old boy, who acquired disease of the hip while convalescing from measles. Rectal examination showed an abscess on the inner wall of the acetabulum, about the size of a hen's egg. The presence of this abscess explained the pain on defæcation of which the patient had complained for some time. On resection, a tubercular sequestrum, the size of a bean, was found in the floor of the acetabulum.

(3) A 5-year-old boy, with spondylitis. On rectal examination, an abscess, the size of a walnut, was felt on the inner surface of the acetabulum. A resection showed a perforation of the acetabulum into the pelvis.—*Cincinnati Lancet-Clinic*, Jan. 27th, 1887.

**A Case of Strangulated Hernia, producing Acetonæmia in a Diabetic Subject. Herniotomy under Cocaine. Coma and Death.**—Dr. Railton and Mr. Southam publish this interesting case. The patient, a woman æt. 56, came under the care of Dr. Railton at the Manchester Clinical Hospital for Women, in November, 1886, complaining of constant thirst and a rapid loss of flesh. Her urine was found to contain a considerable amount of sugar, but no albumen. Her weight was then 113 lbs. 6 ozs. She attended regularly up to Feb. 17th, 1887. The amount of sugar in the urine varied, sometimes being entirely absent. Her weight steadily diminished: on Feb. 17th it was 95 lbs. No albumen was seen; her breath was invariably free from the odour of acetone; and there was an entire absence of claret-colour reaction in the urine when tested with perchloride of iron for aceto-acetic acid.

On Feb. 23rd, Dr. Railton was asked to see her at her own home. As he was about to enter the bedroom, he noticed, for the first time, the acetone smell, which pervaded the air to such an extent that it was perceptible outside the bedroom door. It appeared that the patient had vomited everything since the 21st; and complained of pain in the left groin. On examination, a small hernia was found, evidently strangulated. She had suffered from hernia for some years, but as it caused no trouble she had not called attention to it. The urine, when tested, was now found to contain aceto-acetic acid, but no albumen. Herniotomy was performed under the influence of cocaine, and was almost painless. The next morning the patient appeared to be progressing satisfactorily; but in the evening she suddenly became comatose, and died the following morning, thirty-six hours after the operation.

An hour before death the nurse observed that the entire surface of the body became of a bright red colour, like a boiled lobster; and just before death the hands and feet became perfectly black for the space of about five minutes.

At the post-mortem examination, the wound was found to be healthy, and showed well-marked signs of repair. The smell of acetone was strongly perceptible in the brain, and, though faint, was recognisable in the liver. The brain and kidneys appeared healthy; the heart was flabby, with a pale yellow colour, and right enlargement; the lungs were congested ; and the liver presented some pale fatty-looking patches, but was otherwise healthy.

This case was a mild form of diabetes, slow-moving with spontaneous remissions. Some consider cases of this class to be mere glycosuria rather than true diabetes. It is interesting to note that, even in these cases, the danger of a shock to the nervous system is almost equally as great as in the more typical forms. In this case the acetonæmia was doubtless due to the shock produced by the strangulation of the bowel, and was not perceptibly increased by the operation.—*The Medical Chronicle*, April, 1887.

**The Surgery of the Pancreas.**—Dr. Senn, in an excellent and detailed series of papers in the *International Journal of the Medical Sciences* for July and October, 1886, and January, 1887, gives the results of his experimental and clinical researches on the pancreas, from a surgical point of view. In concluding, he submits the following propositions:

(1) Restoration of the continuity of the pancreatic duct does not take place after complete section of the pancreas.

(2) Complete extirpation of the pancreas is invariably followed by death, produced either by the traumatism or gangrene of the duodenum.

(3) Partial excision of the pancreas, for injury or disease, is a feasible and justifiable surgical procedure.

(4) Complete obstruction of the pancreatic duct, uncomplicated by pathological conditions of the parenchyma of the organ, never results in the formation of a cyst.

(5) In simple obstruction of the pancreatic duct, the pancreatic juice is removed by absorption.

(6) Gradual atrophy of the pancreas, from nutritive or degenerative changes of the secreting structure, is not incompatible with health.

(7) Physiological detachment of any portion of the pancreas is invariably followed by progressive degeneration and atrophy of the glandular tissue.

(8) Extravasation of fresh normal pancreatic juice into the peritoneal cavity does not produce peritonitis, but is rapidly removed by absorption.

(9) Crushed or lacerated pancreatic tissue is removed by absorption, provided the site of operation remains aseptic.

(10) Complete division of the pancreas by elastic constriction is never followed by restoration of interrupted anatomical continuities.

(11) Limited detachment of the mesentery from the duodenum, as required in operations upon the pancreas, is not followed by gangrene of the bowel.

(12) In all operations upon the head of the pancreas, the physiological connection of the peripheral portion of the gland should be maintained by preserving the integrity of the main pancreatic duct.

(13) Partial excision of the splenic portion of the pancreas is indicated in cases of circumscribed abscess and malignant tumours, in all cases where the pathological product can be removed completely without danger of compromising pancreatic digestion or inflicting additional injury upon important adjacent organs.

(14) Ligation of the pancreas at the point or points of section should precede extirpation, as a prophylactic measure against troublesome hæmorrhage and extravasation of pancreatic juice into the peritoneal cavity.

(15) The formation of an external pancreatic fistula by abdominal section is indicated in the treatment of cysts, abscesses, gangrene, and hæmorrhage of the pancreas due to local causes.

(16) Abdominal section and lumbar drainage are indicated in cases of abscess or gangrene of the pancreas where it is found impossible to establish an anterior abdominal fistula.

(17) Thorough drainage is indicated in abscess and gangrene of the pancreas, with diffuse burrowing of pus in the retro-peritoneal space.

(18) Removal of an impacted pancreatic calculus in the duodenal extremity of the duct of Wirsung, by taxis or incision and extraction, should be practised in all cases where the common bile-duct is compressed or obstructed by calculus, and death is threatened by cholæmia.

(19) In such cases, the principal source of danger—extravasation of bile into the peritoneal cavity—should be avoided by preliminary aspiration of the dilated bile-ducts, accurate closure of the visceral wound with fine silk sutures, and absolute physiological rest of the organs of digestion during the time required in the healing of the visceral wound.

**Paralysis due to the Compression of the Spinal Cord.**—Dr. Strumpnell, of Erlangen, has recently made some interesting observations concerning the paraplegia caused by compression of the spinal cord, especially when the latter is caused by accumulation of pus from diseased vertebræ, or by other affections of the bony covering of the cord.

Paraplegia is frequently caused by myelitis, which may be produced by various morbid lesions, among which is compression. Compression, however, may cause paraplegia by mechanical action without the development of any inflammatory process. These are the cases where, after death, no degeneration of the cord is found; and also those in which, after removal of the compressing material, the paralysis disappeared, and motion was as good as ever.

In Pott's disease with paraplegia due to effused fluids, these are sometimes absorbed with surprising rapidity, when the curative inflammation has resulted in perfect immobility of the parts.

The point to which Strumpnell has called attention is of great practical importance, both as regards prognosis and treatment.—*Medical and Surgical Reporter*, Feb. 19th, 1887.

W. J. PENNY.

**Cocaine in Lithotrity.**—In *El Siglo Medico* for March, 1887, Dr. E. Suender gives the following record: Dr. Robert Weir, of New York, first performed lithotrity, using hydrochlorate of cocaine as a local anæsthetic, in February, 1885: he injected into the bladder 10 grammes of a 4 per cent. solution, and fifteen minutes after proceeded to crush a stone of 2 centimetres in diameter; the operation was satisfactorily completed in from eight to ten minutes. Dr. Brungs, of Tubinga, shortly afterwards injected 40 grammes of a 2 per cent. solution into the bladder, and 10 grammes of the same solution into the urethra. After waiting six or eight minutes he completely filled the bladder with warm water; he then crushed a stone of more than 2 centimetres in diameter, the detritus weighing 4 grammes. The operation lasted twenty-two minutes, the patient having felt no pain whatever during the crushing.

In December of the same year Dr. Dubac communicated to the Medical Society of Paris the result of an operation he had performed, under cocaine, on a patient who had been operated upon both under chloroform and without an anæsthetic. The quantity of cocaine used was 1 gramme in 40 of water; the result was satisfactory, but the patient expressed his preference for chloroform.

Dr. Watson, of Boston, injected 28 grammes of a 4 per cent. solution, and crushed a stone the *débris* of which weighed 4½ grammes. In fifteen minutes—the operation being then nearly completed—the patient complained of sharp pain, and there was spasmodic contraction of the bladder. A second injection of cocaine was introduced, and the operation completed painlessly.

Dr. Boeckel, of Strasburg, with the object of completing the crushing of a stone which operation he had commenced five days previously under chloroform, injected a 4 per cent. solution of cocaine; the sensibility of the bladder was scarcely diminished, and the operation was therefore suspended. Two days later he injected a 10 per cent. solution, when complete anæsthesia, lasting twelve minutes, was obtained. Five days after, he injected a 15 per cent. solution, when he obtained perfect insensibility of the bladder, lasting nearly half an hour.

Dr. Delefosse used a 3 per cent. solution, of which he injected 50 grammes: in ten minutes the patient became pale and complained of nausea; in five minutes more he vomited. The passage of the lithotrite was painful; but the crushing was well borne for eight minutes, when the patient complained of sharp pains in the bladder, and the completion of the operation was postponed.

Dr. Calleonzis, of Athens, injected into the bladder 100 grammes of a 5 per cent. solution, equal to 5 grammes of the hydrochlorate: after twenty minutes, without drawing off the injection, he began to crush a hard calculus. The operation lasted twenty-four minutes; the *débris* weighed 6 grammes.

Dr. E. Suender (the writer), on the 1st of December, 1886, operated upon a man 60 years of age. He injected 30 grammes of a 5 per cent. solution: after waiting twenty-five minutes, he introduced the lithotrite and crushed the stone. The operation lasted six minutes, and was painless throughout.

On the 26th December he operated on a man, 20 years of age, who had suffered from stone for four years. He injected 40 grammes of a 5 per cent. solution: in twenty-five minutes the patient complained of loss of sensation all over the body, and great heat about the lower extremities; he then vomited some yellowish liquid. The pulse and temperature remained normal. All the symptoms disappeared when the bladder was emptied. The *débris* of the stone—oxalate of lime—weighed 18 grammes.

Dr. Bignon, of Lima, has arrived at the following conclusions:

(1) The physiological effects of cocaine are transitory, if taken in quantities of from 30 to 50 centigrammes, in doses of 5 centigrammes every hour.

(2) The alkaloid acts principally upon the urinary secretions, impeding the elimination of the products of oxidation, and producing slight symptoms of uræmic poisoning.

(3) In large doses it produces anuria.

(4) The action upon the kidneys passes off in two or three hours, and is followed by diuresis.

(5) Cocaine produces toxic effects in an indirect manner—that is to say, by suppressing the elimination of toxic products, uræmic poisoning results. J. Bain Sincock.

### GYNÆCOLOGY.

**The Operation of Shortening the Round Ligaments.**—An excellent paper on this subject, read before the Baltimore Academy of Medicine by Dr. Thomas Ashby, ended with the following summary of conclusions:

(1) The round ligaments are designed to hold the uterus in its axis in the pelvis, and to draw the fundus of the organ towards the symphysis pubis. They have little if any sustaining power in preventing procidentia, except in extreme cases of descent, where the organ has escaped outside the vulva. Exterior displacements of the uterus can only take place when the round ligaments have been relaxed or stretched by prolonged tension.

(2) Shortening the round ligaments is a practical method by which the uterus may be lifted into its normal axis, and be retained in position by a restoration of its normal supports.

(3) The operation is admissible in all cases of posterior displacement where the uterus is not fixed by adhesions, but perfectly movable in the pelvis, and where other methods of support are not of service.

(4) The operation can prove of little value in cases of procidentia, except when employed in conjunction with other methods instituted to overcome this form of displacement.

(5) The operation can be easily performed by one who is familiar with the anatomy of the parts. It is almost devoid of danger if ordinary safeguards are employed.

(6) In the class of cases to which it is limited, the benefits secured are striking and important.—*New York Medical Journal*, Feb. 12th, 1887. W. J. Penny.

### LARYNGOLOGY.

**Influence of the Laryngoscope on the Diagnosis of Extra-laryngeal Affections.**— Paul Koch (extract from *Journal of Laryngology and Rhinology*, Feb., 1887). The author points out how great is the assistance afforded by the use of the laryngeal mirror in the diagnosis of general diseases; and after referring to laryngeal phthisis, he asserts that ulceration of the larynx in typhoid fever is of much commoner occurrence than it is thought to be. In acute articular rheumatism, although the crico-arytenoid joints are only exceptionally affected, the laryngeal dyspnœa sometimes met with in this disease is explainable by the discovery of round painful swellings over the arytenoid cartilages. He refers to the laryngeal neuroses of locomotor ataxy; finally he refers to hysterical throat affections, and considers that they can be explained only on the assumption of cortical motor centres, which are bilateral and independent of the centre of speech, which is only unilateral.

[There cannot be a doubt, that the laryngoscope is not used as frequently as it ought to be in general medical practice, mistakes being made, owing to this neglect, which would be entirely avoided, if only all practitioners would use this instrumental means of diagnosis, as they do the ophthalmoscope.]

**Primary Syphilis of the Tonsils.**—Dr. Malin, *Norsk Magazin for Lægeridenskaben*, Oct., 1886, describes two cases of chancre of the tonsil. The first was in a man, aged 54, whose left tonsil was affected; the second in a woman, aged 56, whose right tonsil was the seat of the disease. In both cases typical secondary symptoms were seen, and the characteristic ulceration of the surface of the chancre ensued. The infection is supposed to have been caused by foul drinking-vessels.

Thomaschewski, *Wiener Med. Presse*, Nos. 30—35, 1886, relates particulars of two cases in which one tonsil was thus affected—being hard, much enlarged, the colour of raw flesh, and ulcerated. Pain in swallowing was slight; the cervical glands were swollen and hard, and a macular and papular eruption developed. The external inguinal glands were affected more than the internal ones.

[The diagnosis of these cases is often very difficult; and an obstinate tonsillitis, with some superficial ulceration, is looked on and treated as a follicular inflammation with cheesy exudation, with, of course, no great good being done. The induration of the tonsil and the large, unilateral, hard and usually painless, swelling at the angle of the jaw, and also the swollen, hard, cervical glands, are diagnostic. Of course, the secondary rash puts an end to one's difficulties. I have lately had a case of chancre of the left tonsil in a young girl, in which the affection had previously been diagnosed as a simple tonsillitis, but in which the characteristic ulcer, followed by cicatrisation, leaves no room for doubt as to its syphilitic origin, although the rash was of a most evanescent character. Anti-syphilitic remedies speedily cured the condition.]

**The Treatment of Nasal Diphtheria.**—A. C. Reierson (extract by H. Mygind, *Journal of Laryngology and Rhinology*, Jan., 1887). The author, believing that the pituitary membrane forms a nidus for micrococci more easily than almost any other mucous membrane, and finding that the treatment of the affection by the injection of antiseptic liquids through the nostrils, or by the removal of the false membrane by forceps, is often very unsatisfactory, suggests the use of bougies in order to apply drugs to the affected parts. The bougies are made thus:

Cocain. Hydrochlor., gr. $\frac{1}{5}$.
Acid. Boracic., grs. xv.
Amyli,
Gummi Arab. pulv., $\overline{aa}$ gr. iss.
Glycerin., q.s.

The size is that of the usual nasal bougie, and for children shorter, but of the adult diameter, the amount of cocaine and boracic acid in each being less.

One is to be applied to each nostril, and pushed along the inferior meatus until it reaches the naso-pharynx. They melt in an hour; and if relief be not experienced after syringing the nostrils, they may be again introduced.

BARCLAY J. BARON, M.B.

**Deformities of the Nasal Septum.**—Professor Bosworth, in an interesting paper read before the New York Academy of Medicine, describes the varieties of deformity of the nasal septum. After alluding to deformities caused by deflection of the whole or parts of the septum, he describes a condition which he considers to be generally due to traumatism. This consists briefly of a prominent ridge running along the sutural lines, and which may be classed under three heads, in their order of frequency, as follows:

First, that in which the ridge is found along the line of junction between the vomer and the palatal process of the superior maxillary bone.

Second, that in which the ridge is found extending along the junction of the cartilage of the septum and of the vomer, and ending abruptly at the junction of the upper border of the septal cartilage and the perpendicular plate of the ethmoid.

Third, that in which the ridge extends along the whole anterior edge of the vomer, including its union with the cartilaginous septum, as well as perpendicular plate of the ethmoid bone. This deformity is most frequently confined to one side, but may be developed on both sides of the same suture. It consists in a sharp-pointed spur or ridge, as it were, projecting on one side of the septum, with, in no case, a corresponding depression on the other. It is therefore not a deflection in any sense of the word. He knows no better way of explaining its existence than to regard it as a chronic arthritis, or perhaps a deforming arthritis as the result of an injury.

In the treatment of these deformities, the object in view has been to restore normal nasal respiration. Various operations resorted to by Blandin, Adams, Steele of St. Louis, Jarvis of New York, Roberts of Philadelphia, Chassaignac, Gross, and Ingalls are referred to; but Professor Bosworth states that, in his hands, these various operations have failed to meet the requirements of a large majority of cases. He consequently invented a saw, with which, after anæsthetising the parts with cocaine, the projecting ridge is sawn straight off, as "we saw a board out of a log," the object being to reduce the septum as far as possible to its normal outlines. Professor Bosworth has operated on 166 cases; in one case only was the septum cut through.

Forty-five cases were operated on for hypertrophic rhinitis, of which 32 were cured and 13 relieved. The symptoms of these cases were briefly those of nasal stenosis, with mouth breathing. Dr. Bosworth states that mouth breathing is never a habit; it is a necessity, due to the fact that the individual cannot get air enough through the nose.

Twenty-one cases were done for simple chronic rhinitis. The prominent symptoms of this class were, especial liability to cold, nasal stenosis, snuffling, and watery discharge from the nose. The majority were cured by the operation; a few cases of long standing required a little after-treatment.

Five cases of laryngitis were treated, with 3 cures and 2 markedly relieved.

There were 18 cases of bronchitis, of which 8 were cured and 10 relieved.

Five cases of simple irritative cough were all cured.

Thirteen cases of hay fever, with 5 cures.

Eleven cases of hay fever and asthma, with 5 cures.

Twelve cases of asthma alone, with 6 cures.

Two cases of rose-cold; both cured.

Two cases of rose-cold and asthma; both cured.

The special points to which attention is called are: First—that a nasal stenosis due to obstructive lesion must necessarily be followed, sooner or later, by hyperæmia of the membrane beyond the point of obstruction; this occurring, first in the nose, and secondly in the larynx, trachea, and bronchial tubes.

Second—a hyperæmia of the nose is liable to be followed by hyperæmia of the air-passages beyond, as the result of an intimate physiological connection between the two, both deriving their vaso-motor control probably from the same central ganglia; this hyperæmia, either in one location or in the other, tending towards the development—first, of catarrhal inflammation with hypertrophy, as shown in the cases of laryngitis and bronchitis dependent on nasal disease, and only relieved by treatment of the nose; and, second, in those cases in which complete vaso-motor paralysis develops in the nose, giving rise to hay fever, which, sooner or later, is followed by vaso-motor paralysis in the bronchial mucous membrane, causing true asthma.

Twenty-four of the cases treated suffered from more or less impairment of hearing, and were all benefited by the operation.

In conclusion, Professor Bosworth expresses his belief that climate has very little to do with causing a morbid condition of the upper air-passages, but has much to do with aggravating the condition when it exists. He believes that a large majority of cases are primarily due to injury, as a rule, inflicted during childhood.—*The Medical Record*, Jan. 29th, 1887.

W. J. PENNY.

**On the Value of Lactic Acid in the Treatment of Phthisis Laryngea.**—Dr. R. Masini, quoted by *Revue mensuelle de Laryngologie*, Feb., 1887. The author has used lactic acid, as recommended by Krause and Jélinek. At first, in 20 per cent. solution, which caused a little cough, a sensation of burning, and intense redness of the parts touched with it. A 40 per cent. solution caused a feeling of constriction, and also ulceration; and, in spite of the use of cocaine, the patients were scarcely able to bear the treatment because of the irritation set up by it, and in one case hæmoptysis occurred. In spite of this, Dr. Masini used the acid in 80 per cent. solution, as recommended by Krause; the result being that, although cocaine was employed previously to the acid, such violent spasm of the glottis supervened, that artificial respiration had to be performed in order to keep the patient alive.

The author does not, therefore, like the lactic acid treatment, and much prefers an ethereal solution of iodoform.

[I have not yet tried lactic acid in the treatment of this terrible disease; but, from what I have repeatedly seen when using solutions of nitrate of silver in the larynx, I can quite believe that a strong acid like lactic acid would set up great irritation, and, in some patients, dangerous spasm. It is also difficult to understand what especial effect, other than a cauterising one, the substance can exert on parts infiltrated with tubercle. The iodoform treatment I find very useful: either powdered iodoform insufflated, or a saturated solution in ether, applied with a brush or a dropper. Menthol—20 per cent. solution in olive oil—dropped on to the affected parts, also seems to quiet pain.]

**Tuberculous Growths in the Larynx.**—R. Ariza (extract by R. de la Sota, in *The Journal of Laryngology and Rhinology*, Jan., 1887). The author describes two cases of the polypoid form of laryngeal tuberculosis. One was a man, 50 years old, with a tumour attached by a broad base to the inter-arytenoid laryngeal wall; the other was a young lady with several growths on the same region, and a long thin polypus, which during speaking was concealed beneath the vocal cords, but during coughing protruded between them. Both patients suffered from phthisis pulmonalis.

There is another species of laryngeal tuberculosis which Dr. Ariza calls the "hypertrophic," as distinguished from the "polypous." In this form the enlargements are diffuse, cover a considerable space, and are lost gradually in the neighbouring parts. The usual seats of this affection are, the mucous and submucous tissue of the ventricular bands, and inter-arytenoid space: its colour is deep red, with some whitish spots.

Tubercular polypi are characterised, according to Ariza, by—

(1) Greyish spots, indicative of epithelial destruction.

(2) The facility with which they ulcerate, on the slightest traumatic or caustic action.

(3) Their softness and yielding nature.

(4) Being generally single.

[Tuberculosis of the larynx is so often destructive in its action, after the stage of œdema and infiltration has passed, that one is apt to picture to oneself the turban-like epiglottis, the large pyriform swellings of the arytenoids and ary-epiglottic folds during the stage of infiltration; and the ulcers of the epiglottis, arytenoids and vocal cords, in the stage of breaking down, as being the only pathological appearances revealed by the laryngoscope. I regard it, therefore, as of no small value, that we should be reminded that there are outgrowths in chronic phthisis laryngea as well as in chronic laryngitis and tertiary syphilis; and all who have had much experience of laryngoscopy will agree with me, that it is sometimes a matter of considerable difficulty to say whether we have to do with a primary phthisis of the larynx, or with some syphilitic affection of the organ, supposing the possibility of syphilis to be denied.]

**Intubation of the Larynx.**—R. Norris Wolfenden, *Journal of Laryngology and Rhinology*, Jan., 1887. The author describes the set of instruments used in this operation as consisting of a gag, five laryngeal tubes, an applicator, extractor, and a gauge. The tubes range from 1¾ in. to 2½ in., the calibre being ⅛ in. to ¼ in. in the largest, and not more than half this in the smallest. Silk thread is passed through an eye in the upper end of the tube. Jointed obturators hold them during introduction, and into their upper extremity is screwed the applicator. A sliding tube fits on to the stem and covers it, so that on pushing it forward with the thumb, the obturator is released and can be withdrawn, and the tube left in position. The tube is extracted by putting the extractor into it closed, opening the blades of which it is composed, and withdrawing it and the tube.

The operation is thus performed:

The patient faces the operator; the head and arms are held by an assistant, and the mouth is kept open by the gag. The left index finger, passed across the base of the tongue, hooks up the epiglottis, and serves to guide the tube into the larynx. The handle of the applicator is held towards the sternum until the pharyngeal wall is reached; the point is then directed downwards and forwards along the inner edge of the index finger, the handle being carried high, and the tube directed well forwards to avoid passing it into the œsophagus. Ten to twenty seconds are said to be sufficient for this part of the operation, a characteristic cough showing that the tube is in the larynx, easy respiration being established when the obturator is withdrawn. Now cut one end of the silk thread; pass one finger behind the epiglottis, and hold the tube while the thread is removed. If the tube be stopped by secretions, it must be withdrawn. The withdrawal is said not to be easy: pressure of the larynx backwards and upwards with the left thumb facilitates it; but an anæsthetic may be necessary.

It has been used almost entirely in cases of diphtheria and croup, and Dr. Cheatham (*American Practitioner and News*, Nov. 13th, 1886) gives statistics of 95 cases, of which 28 recovered, or 29·47 per cent. Tracheotomised cases averaged 18·95 per cent. of recoveries. Dr. Wakham has had 29 recoveries in 96 cases, or 30.2 per cent.

Dr. Strong has performed it successfully in a case of acute catarrhal laryngitis.

BARCLAY J. BARON, M.B.

**"Tubage" as a Substitute for Tracheotomy.**—After describing the mode of introducing the tube, the author points out that Professor Stoerk, of Vienna, has shown that the tube rests on the true, and not on the false, vocal cords, as O'Dwyer imagined. The objections to its use are:

(1) Difficulty of introduction in some cases: in several instances the tube has passed into the œsophagus.

(2) Troublesome cough with rejection of the tube, in many cases again and again. O'Dwyer attributes this to small size of the tube employed; but however this may be, it would seem to preclude "tubage" without risk, unless the patient could be under the constant surveillance of the medical attendant.

(3) When the tube is of sufficient size, it produces small ulcers in its neighbourhood.

(4) In several cases it has been swallowed, and found in the stomach at the autopsy.

(5) So far as statistics go, at present the number of recoveries is from 27 to 28 per cent. in cases where intubation of the larynx, and from 19 to 25 per cent. where tracheotomy, was performed.—*Le Progrès médical*, April 20th, 1887.

J. MICHELL CLARKE, M.B.

### THERAPEUTICS.

**Treatment of Dilatation of Stomach.**—M. Mathieu describes the mode of treatment that he has found most successful in this disorder. Dilatation of the stomach is not a disease, but a symptom arising in different classes of cases arranged under the following heads:

(1) Dilatation following organic disease and contraction of pylorus.

(2) Dilatation from degeneration of the gastric muscular fibre.

(3) Dilatation in debilitated persons, more marked in the stomach than in any other part of the alimentary canal, because of the large size of the stomach, and of its supporting the whole mass of food at each meal.

(4) Dilatation from atony of muscle in neurasthenia, alternating at first with other symptoms, but finally predominating over and persisting beyond these.

In the first two varieties there is obviously no cure; relief may be afforded by breaking up the food as much as possible, and taking very small quantities at a time.

Under (3) and (4) the indications for treatment are:

(1) To avoid overloading the stomach; if the stomach receives suddenly a large amount of food, the mere weight of this will exert considerable traction on its walls.

The total quantity of food taken is to be reduced: if this cannot be done, the meals must be diminished in size and increased in frequency; at the same time, the food must be thoroughly masticated. No vegetables or green fruits of any kind, except a little potato. Light (foreign) beers are advantageous; it is better to forego wine, and no alcoholic drink is to be taken except at meal-times.

M. See recommends, when there is pain, the use of hot drinks—weak hot tea in the mornings, and hot water with a very small amount of spirits, if desired, at night. No cold or iced drinks should be taken.

(2) To secure a regular evacuation of the bowels, since constipation is present in most of these patients, a powder composed of equal parts of magnesia, cream of tartar, and precipitated sulphur, is recommended; a teaspoonful in coffee, three times a day before meals. The patient will soon find out the right dose for himself. Another very good plan is a small enema of cold water each morning, with two or three teaspoonfuls of glycerine by the mouth.

(3) To promote general well-being, the author thinks that the cold douche or shower-bath is most efficacious.

(4) To restore the normal muscular tone to the stomach, he considers that ipecacuanha is the best remedy, given in the form of 1 to 2 grains in powder, in capsule, every morning. Smaller doses may be used for susceptible persons. Reduction of the stomach area of resonance, under the influence of ipecacuanha, is the most important element in the prognosis: he thinks that this remedy causes a contraction of the muscle fibres, comparable to the contraction of the heart-muscle from digitalis. Should the dilatation of the stomach still remain the same after the above treatment has been persevered with some little time, the inference may be drawn that the muscle is degenerated.

(5) To relieve pain, hot fluids are useful; and chloroform water or cocain, gr. $\frac{3}{4}$ to $1\frac{1}{2}$.—*Le Progrès médical*, Feb. 12th, 1887.

**On the Treatment of Neuralgias.**—The special action of aconitine is to lower or suppress the functions of the sensory nerves; while at the same time it produces anæsthesia, diminishes the calibre of the capillaries, and lowers the temperature. It is especially efficacious in facial neuralgias—above all, in those attended with congestion; and in some other so-called rheumatic neuralgias, from cold. It is generally useful in catarrhal affections, and can be administered in very small doses at convenient intervals.

Aconitine is useful in other diseases besides neuralgia: it has given excellent results in hemicrania, migraine, pleurodynia, and in rheumatic pains in the joints.

Its action is certain and exact; but on account of its potency, it is necessary to give it in small doses at considerable intervals: one must also be sure that the preparation is a good one, and constant in strength.

Neuralgia has often a marked periodicity: to combat this complication, Dr. Moussette, of Paris, has composed pills, each exactly containing $\frac{3}{1000}$ grain aconitine, combined with quinine. Until the susceptibility of the patient is known, it is as well to give one pill at morning, mid-day, and evening, for the first day. If there is no marked effect from this, the dose is gradually increased by one pill daily, until six are taken in the twenty-four hours: these may be continued until relief from pain is obtained; but it is as well not to go beyond this quantity, unless in exceptional cases at any rate. If any diarrhœa comes on, the dose is to be diminished.

In the Paris hospitals the administration of aconitine in this way has given very satisfactory results.—*Le Progrès médical*, Feb. 12th, 1887.

**On some Therapeutical Applications of Chloral.**—Grave inconveniences and even danger result from the administration of opium and its derivatives to patients suffering from albuminuria; at the same time, there is often insomnia, teasing cough from pulmonary œdema, and severe renal or sciatic pains, that demand the use of a sedative or narcotic. A case is here quoted of a woman who suffered from albuminuria during an attack of erysipelas following amputation of the breast for scirrhus. She suffered from most intense sciatica, and a troublesome cough that prevented sleep. It could not be determined whether the albuminuria was of old standing. She took 15 grains of chloral hydrate, and passed a good night. After this she took 30 to 45 grains daily, for several months, without interruption. She recovered from the effects of the operation and the erysipelas. When seen three years after, there was no return of the cancer; and the albumen which for one year had been present in the urine in the large amount of 10 grammes per litre, now oscillated between 2 and 3 grammes per litre. She still took the chloral from time to time when the sciatica returned, but her general health had much improved. This case goes to show that chloral may be given without fear of accumulation to albuminuric patients for long periods.

The author then quotes two cases of Dr. Wilson's from the *British Medical Journal*, where chloral, at first given for its narcotic effect, produced first amelioration, and finally cure of the albuminuria. In renal eclampsia chloral is undoubtedly efficacious.

In whooping-cough it is claimed that chloral gives better results than any other drug, both in shortening the course and lessening the severity of the disease. In children, from the age of 5 years and upwards, 5 to 6 grains may be given at intervals of eight hours. For younger children a dose of 3 grains is enough; milk disguises the taste very well. It should never, of course, be prescribed if bronchitis or bronchopneumonia be present.

In chorea, excellent effects result from the administration of 15 to 22 grains daily to children below five years of age, and of 30 to 37 grains to older ones; stopping short at a small degree of the physiological effect of the remedy, so as to allow the child to be awake part of the day.—*Le Progrès médical*, March 20th, 1887.

J. MICHELL CLARKE, M.B.

**Cocaine in Whooping-cough.**—L. E. Hall (*New York Medical Journal*, Oct., 1886) comes to the following conclusions:

(1) It must be used with great care in young children.

(2) The spray is not to be recommended, as the quantity administered is uncertain.

(3) No solution stronger than 4 per cent. ought to be administered to children less than two years old.

(4) It never is of any great value.

(5) Chloral controls the symptoms set up by its use.

A. P. Garnett (*Journal of the American Medical Association*, Oct., 1886) uses a 6 per cent. solution of cocaine in chloroform, and allows a child to inhale 10 minims of the solution from a wineglass made warm by tepid water, the nostrils being kept closed. The dose to be repeated every four hours. It relieves paroxysms, but does not cut short the disease.

BARCLAY J. BARON, M.B.

**Scraping in Surgery.**—Mr. Pridgin Teale strongly advocates the use of the sharp spoon, originally introduced by Volkmann, and improved by Sir Joseph Lister. Mr. Teale recommends scraping for indolent sores and sinuses leading to extensive cavities, after laying open the latter. In cases of scrofulous glands there is a wide field for the scraper, not only for dealing with sinuses and suppurating cavities, but for scooping out and eradicating damaged and caseating glands. It is useful also in cases of lupus and lupoid sores. In buboes and carbuncles, after incising the affected parts, Mr. Teale employs the scraper freely; in the case of carbuncle, the treatment should be supplemented by numerous small incisions into the neighbouring œdematous and inflamed skin. After removing all decayed tissues, the parts should be well soaked with some antiseptic lotion, and dressed with antiseptic materials. In ophthalmic surgery, the use of a fine scraper in cases of tinea tarsi and ulcers of the cornea is gradually becoming a recognised procedure.—*Liverpool Medico-Chirurgical Journal*, January, 1887.

**Treatment of Fissure of the Anus.**—Dr. F. Mendel *(Deutsche Med-Zeitung)* reports the successful treatment of a number of cases of very painful fissure of the anus with nitrate of silver and ointment. The treatment is begun with a thorough application of nitrate of silver, which is rendered in a measure painless by the use of a solution of cocaine. He next makes an application of an ointment composed of boric acid 2 parts, cocaine 1 part, lanolin 20 parts. This is to be applied several times a day with the finger, and kept in place with a small sponge.—*American Practitioner and News*, April 2nd, 1887.

W. J. PENNY.

